# Supplementary material for: Antimicrobial and antibiofilm activity of human recombinant H1 histones against bacterial infections
Source: mSystems. 2024 Oct 29;9(11):e00704-24. doi: 10.1128/msystems.00704-24 (PMC11575268; doi:10.1128/msystems.00704-24)

**Antimicrobial and antibiofilm activity of human recombinant H1 histones against bacterial infections.**

Betsy Veronica Arévalo-Jaimes^a,b^, Mónica Salinas-Pena^c^, Inmaculada Ponte^d^, Albert Jordan^c^, Alicia Roque^d^, Eduard Torrents^a,b^#

^a^Bacterial infections and antimicrobial therapies group. Institute for Bioengineering of Catalonia (IBEC), The Barcelona Institute of Science and Technology, Barcelona, Spain.

^b^Microbiology Section, Department of Genetics, Microbiology and Statistics, Faculty of Biology, University of Barcelona, Barcelona, Spain.

^c^Molecular Biology Institute of Barcelona (IBMB-CSIC), Barcelona, Spain.

^d^Biochemistry and Molecular Biology Department, Universitat Autònoma de Barcelona, Bellaterra, Spain.

Running Head: Combatting bacteria with Human recombinant H1 histones

#Adress correspondence to Dr. Eduard Torrents, [etorrents@ibecbarcelona.eu](mailto:etorrents@ibecbarcelona.eu)

**Fig S1** Circular dichroism spectra of histones H1 subtypes in (**A**) PBS and (**B**) TFE 20%. Represented data are derived for 5 independent measurements.


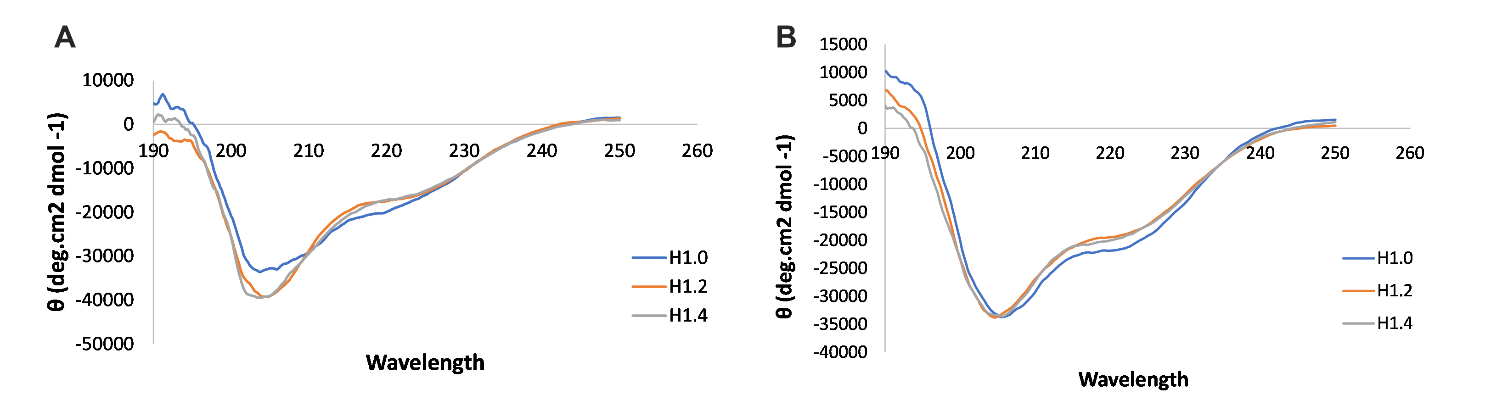

Supplement: Supplemental Material — Figure S1. [file msystems.00704-24-s0001.docx]
